# Activity Dependent Protein Degradation Is Critical for the Formation and Stability of Fear Memory in the Amygdala

**DOI:** 10.1371/journal.pone.0024349

**Published:** 2011-09-22

**Authors:** Timothy J. Jarome, Craig T. Werner, Janine L. Kwapis, Fred J. Helmstetter

**Affiliations:** Department of Psychology, University of Wisconsin, Milwaukee, Wisconsin, United States of America; Alexander Flemming Biomedical Sciences Research Center, Greece

## Abstract

Protein degradation through the ubiquitin-proteasome system [UPS] plays a critical role in some forms of synaptic plasticity. However, its role in memory formation in the amygdala, a site critical for the formation of fear memories, currently remains unknown. Here we provide the first evidence that protein degradation through the UPS is critically engaged at amygdala synapses during memory formation and retrieval. Fear conditioning results in NMDA-dependent increases in degradation-specific polyubiquitination in the amygdala, targeting proteins involved in translational control and synaptic structure and blocking the degradation of these proteins significantly impairs long-term memory. Furthermore, retrieval of fear memory results in a second wave of NMDA-dependent polyubiquitination that targets proteins involved in translational silencing and synaptic structure and is critical for memory updating following recall. These results indicate that UPS-mediated protein degradation is a major regulator of synaptic plasticity necessary for the formation and stability of long-term memories at amygdala synapses.

## Introduction

The activity-dependent synthesis of new protein is commonly thought to be critical for the formation of long-term memories [1]. Consistent with this, numerous studies have found that the transcription of mRNA and subsequent *de novo* synthesis of proteins is critical for the formation of memory in Pavlovian fear conditioning [2]–[4], a widely used paradigm to study the molecular neurobiology of learning [5]. Protein synthesis is considered a necessary step in the transfer of labile short-term memory into a stable long-term memory during the process of memory consolidation [6]. Additionally, recent evidence suggests that the retrieval or recall of established fear memories can induce a second independent phase of protein synthesis which appears to be necessary for memory updating [7] or reconsolidation [8], [9].

The amygdala is believed to be the primary site for the formation and stability of long-term of fear memories [10]. Supporting this, a number of intracellular signaling cascades involved in transcriptional regulation or translational control have been implicated in the formation of fear memories in amygdala neurons [5], [11], [12]. However, it is not currently known if alterations in protein degradation within the amygdala are important during memory consolidation and reconsolidation.

In mammals, the pathway controlling the majority of protein degradation is the ubiquitin-proteasome system. In the UPS, proteins are targeted for degradation through the covalent attachment of a small protein called ubiquitin [13]. Once a polyubiquitin chain has formed, the target protein can then be recognized by S5a, a subunit on the 26S proteasome which captures the target protein for degradation [14], [15]. This system is important for a variety of cellular processes including cell-cycle progression, transcription, apoptosis and more recently has been implicated in synaptic plasticity [16]–[20]. For example, activity-dependent remodeling of the postsynaptic density [PSD] requires new protein synthesis, but evidence now suggests that proteasome-mediated protein degradation is also critical for this same remodeling process [16]. Recently, it has been suggested that protein degradation may also regulate protein synthesis since synaptic stimulation results in a proteasome-dependent reduction in synaptic levels of MOV10, a RNA-induced silencing complex [RISC] factor, which resulted in greater protein synthesis at synapses [21].

Despite accumulating evidence for the role of the UPS in synaptic plasticity, relatively few studies have examined its role in fear memory formation. Recent evidence suggests that protein degradation through the UPS may regulate protein synthesis in the hippocampus during the reconsolidation, but not the consolidation, of fear memory and this may occur through the degradation of PSD scaffolding proteins [22]. However, this finding is in conflict with earlier work showing that protein degradation was critically involved in memory consolidation in the hippocampus [23]. In this case, protein degradation was required for the removal of transcriptional repressors but it is not known if PSD scaffolds were targeted as well. As a result, it remains unclear if protein degradation is required for the consolidation and reconsolidation of hippocampal-dependent fear memories and what potential function it may serve during these processes. Furthermore, no study has examined how protein degradation is regulated when required for consolidation or reconsolidation processes. In order to understand if protein degradation is an important molecular mechanism in long-term memory formation and stability, we need more information about how these alterations in protein degradation relate to established cellular memory mechanisms.

Here we report the first studies looking at the role of UPS protein degradation in the consolidation and reconsolidation of fear memories in the amygdala. We examined whether protein degradation 1) was increased following fear conditioning acquisition and memory retrieval, 2) was triggered by NMDA receptor activity, 3) correlated with established markers of translational regulation, 4) targeted proteins involved in synaptic structure and translational control, and 5) was critical for both the consolidation and reconsolidation processes.

## Results

### Protein degradation is increased in the amygdala following fear conditioning

**Figure 1:**
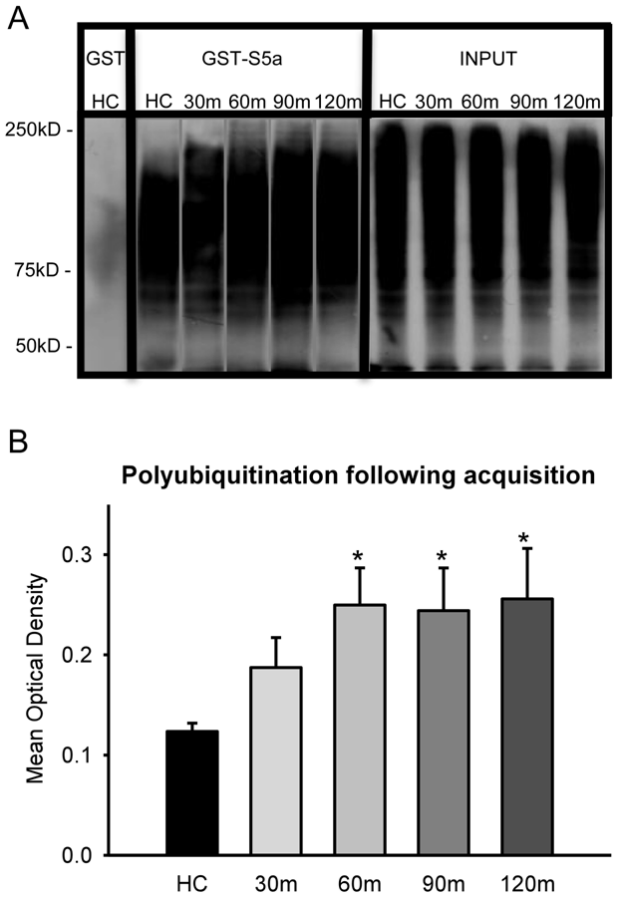
Protein degradation is increased in the amygdala following the acquisition of auditory and context fear memories. [**A**] Amygdala tissue was collected in 30-min increments following fear conditioning [n = 5 per group]. Tissue was purified with GST or GST-S5a and polyubiquitinated proteins pull-downed and exposed to an antibody against ubiquitin. Input represents an aliquot of total ubiquitinated proteins. [**B**] There was a rapid increase in the amount of proteins targeted for UPS degradation following fear conditioning. * denotes p<.05 from homecage [HC] controls.

To determine whether degradation-specific UPS activity is increased in the amygdala during learning and memory consolidation, we trained rats with a standard auditory fear conditioning paradigm in which increased protein synthesis in the amygdala is required for normal memory formation [2]–[4]. Amygdala homogenates were mixed with either GST-S5a agarose or GST-agarose and polyubiquitinated proteins were pulled-down and exposed to an antibody against ubiquitin [Figure 1A]. Fear conditioning training resulted in a robust increase in polyubiquinated protein in the amygdala [Figure 1B]. ANOVA revealed a main effect for time after training [F_(4,20)_ = 2.942, p = .046]. Fisher LSD *post hoc* tests showed that protein degradation was increased within 60-min of acquisition and this increase was sustained for at least another hour relative to naive controls.

**Figure 2:**
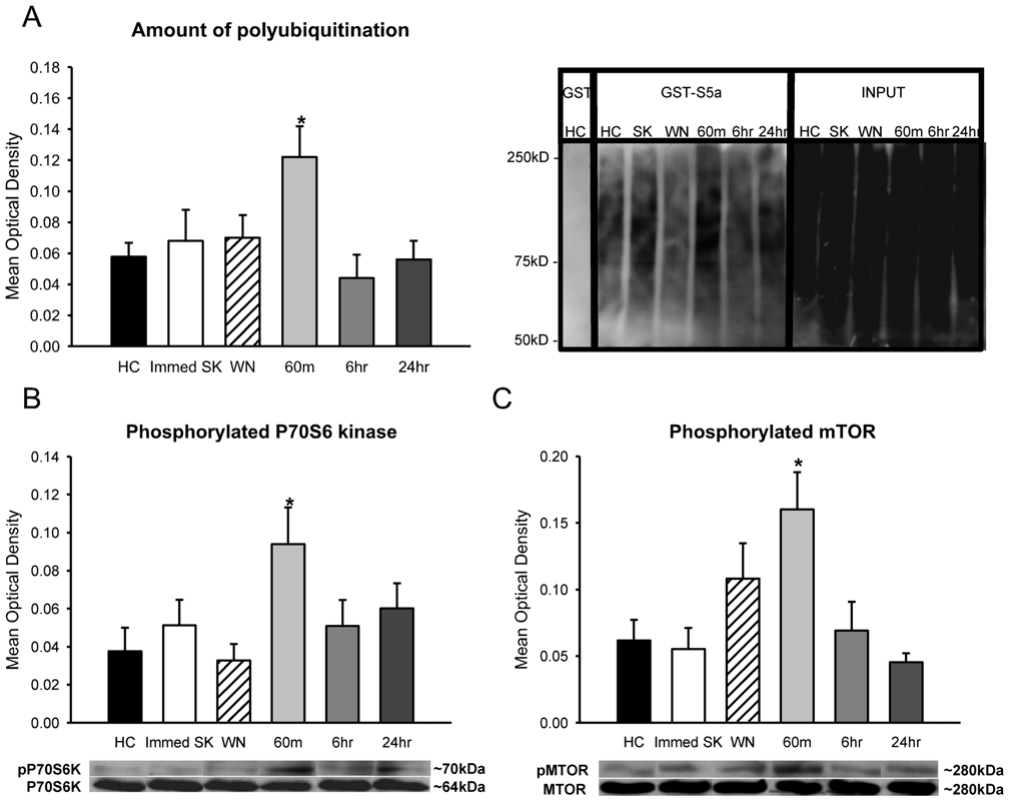
Increase in amygdalar protein degradation is specific to learning and mirrors protein synthesis. Amygdala tissue was collected from naïve animals [HC, n = 8], animals exposed to either the shock [Immed SK, n = 8] or the CS [WN, n = 9], or animals that underwent fear conditioning and were sacrificed 60-min [n = 9], 6- hr [n = 9] or 24-hrs [n = 9] later and tissue was purified with GST-S5a. [**A**] An increase in the amount of polyubiquitinated proteins was only observed 60-min after behaviorally effective training. [**B, C**] Western blots with antibodies against phospho-P70S6 kinase and phospho-mTOR show that increases in protein degradation mirror increases in translational control. * denotes p<.05 from homecage [HC] controls.

Fear conditioning can bidirectionally affect synapse size in the amygdala depending on the CS-UCS contingency during training [24]. In our first experiment, animals were exposed to several pairings of an auditory cue with a footshock in a novel context. It may be possible that the observed increases in protein degradation could be driven by any of these 3 stimuli individually, rather than being driven by the CS-UCS association itself. In order to determine whether the increases in protein degradation we observed were specific to the association of the auditory cue with the footshock, separate groups of animals were exposed to control treatments with the auditory cue or foot shock individually. These controls allow us to directly compare trained animals with those that were exposed to the CS or UCS in the absence of associative learning. Amygdala tissue from the control animals was collected 60-min after acquisition and compared with animals that had the auditory CS paired with the foot shock in normal training. Two additional groups of animals received the normal training protocol and amygdala tissue was collected either 6- or 24-hrs later [Figure S1A] to include time points outside the “consolidation window” of post-training sensitivity to protein synthesis inhibitors [8]. ANOVA revealed a main effect for group [F_(5,46)_ = 2.869, p = .025] and Fisher LSD post hoc tests indicated that the rate of protein degradation was enhanced within 60-min of acquisition relative to naïve controls. This increase was specific to CS-UCS learning, as neither white noise nor shock exposure showed this enhancement. Furthermore, protein degradation returned to baseline levels within 6-hrs of acquisition [Figure 2A]. To confirm this, we immunoblotted samples with an antibody recognizing K48 linked polyubiquitinated proteins [Figure S1B], a degradation-specific polyubiquitin tag [25], [26]. Using planned comparisons, we confirmed that K48 polyubiquitination was enhanced 60-min after fear conditioning relative to all 3 control groups [t_(46)_ = 2.879, p = .006] and the 6- and 24-hr trained groups [t_(46)_ = 2.284, p = .027]. In all cases, the effect size was slightly diminished relative to polyubiquitination detected by S5a. This is consistent with the idea that S5a has the highest affinity for lysine-48 linked chains but can also recognize other linkage sites [27]. Together, this indicates that the increases in protein degradation were specific to the acquisition of the CS-UCS association and fit within the proposed time frame for the completion of the memory consolidation process.

Fear conditioning results in increased protein synthesis and translational regulation in the amygdala [5]. To determine if the pattern of increased protein degradation parallels increases in protein synthesis, we quantified the phosphorylation of two protein kinases [P70S6 kinase and mTOR] related to translational control during the formation of long-term fear memories [12], and used this as an indirect marker of protein synthesis. We observed increases in the phosphorylation of the P70S6 kinase [F_(5,46)_ = 2.533, p = .042; Figure 2B] and mTOR [F_(5,46)_ = 4.496, p = .002; Figure 2C] which both differ significantly from controls at 60-min after training. There were no differences between groups for total P70S6 kinase [F_(5,46)_ = 0.557, p = .732; Figure S1C], total mTOR [F_(5,46)_ = 0.437, p = .820; Figure S1D] or β-actin [F_(5,46)_ = 0.415, p = .836; Figure S1E]. These increases related to translational control closely parallel the observed increases in protein degradation, suggesting a potential overlap between the protein degradation and synthesis processes during the formation of long-term fear memories.

### Increased protein degradation depends on NMDA receptor activity

**Figure 3:**
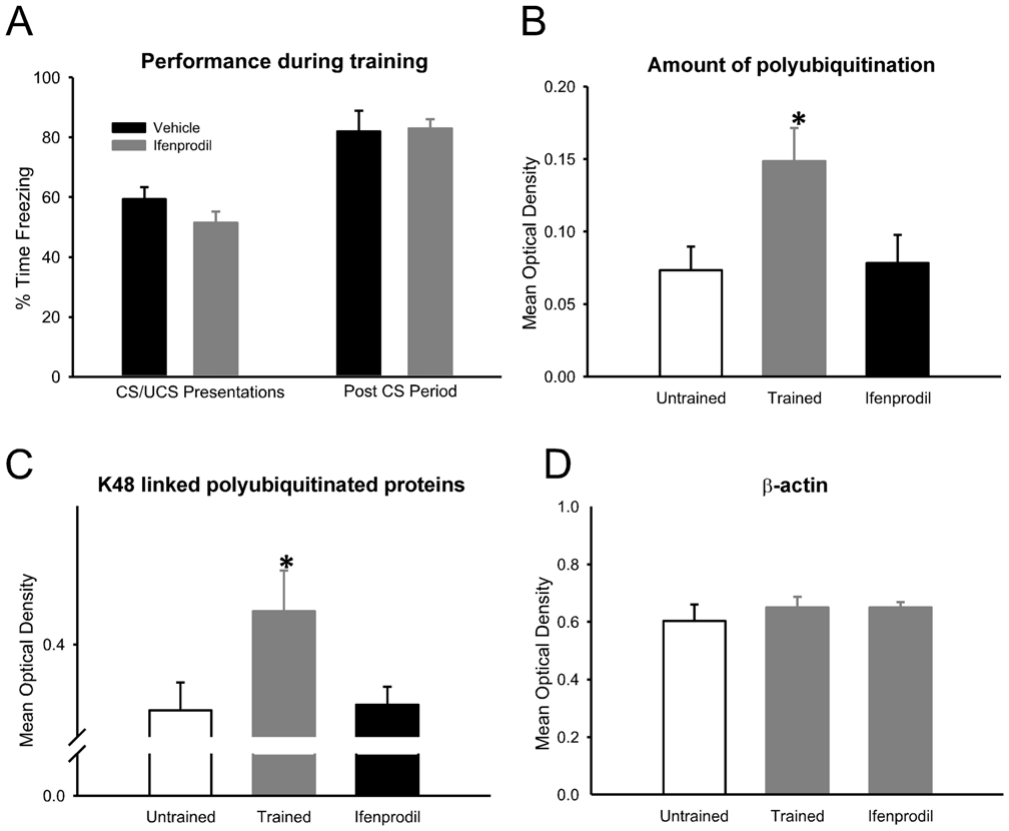
Increase in amygdalar protein degradation is NMDA-dependent. (**A**) Infusions of NMDA antagonist Ifenprodil (n = 8) or vehicle (n = 14) were delivered into the amygdala prior to fear conditioning, and amygdala tissue collected 60-min later and mixed with GST-S5a. Pretraining inactivation of NMDA receptors did not affect performance during training, but (**B**) completely abolished the increase in protein degradation. (**C**) Ifenprodil resulted in a significant reduction of K48-linked polyubiquitination. (**D**) There were no significant differences in β-actin, which was used as a loading control. * denotes p<.05 from untrained controls.

Increases in protein synthesis following fear conditioning are triggered by activation of NMDA receptors [28]. Some evidence exists suggesting that increases in ubiquitin-proteasome activity can also be dependent on NMDA receptors [21], [25], [29]. To see if increases in protein degradation within the amygdala following fear conditioning are related to NMDA receptor activity we infused animals with the NMDA antagonist Ifenprodil prior to fear conditioning at a dose that blocks memory consolidation [28] and collected amygdala tissue for GST analysis [Figure 3A]. ANOVA indicated Ifenprodil completely abolished the degradation increases observed following learning detected both by GST-S5a [F_(2, 19)_ = 4.480, p = .025; Figure 3B] and a K48-linked polyubiquitin antibody [F_(2, 19)_ = 3.428, p = .054; Figure 3C], but did not change β-actin expression [F_(2, 19)_ = 0.580, p = .569; Figure 3D]. These data represent the first *in vivo* link between NMDA receptor signaling and the UPS and suggest that increased protein degradation in the amygdala is induced by a mechanism that is dependent on NMDA receptor activity.

### The UPS targets MOV10 and Shank during memory formation

**Figure 4:**
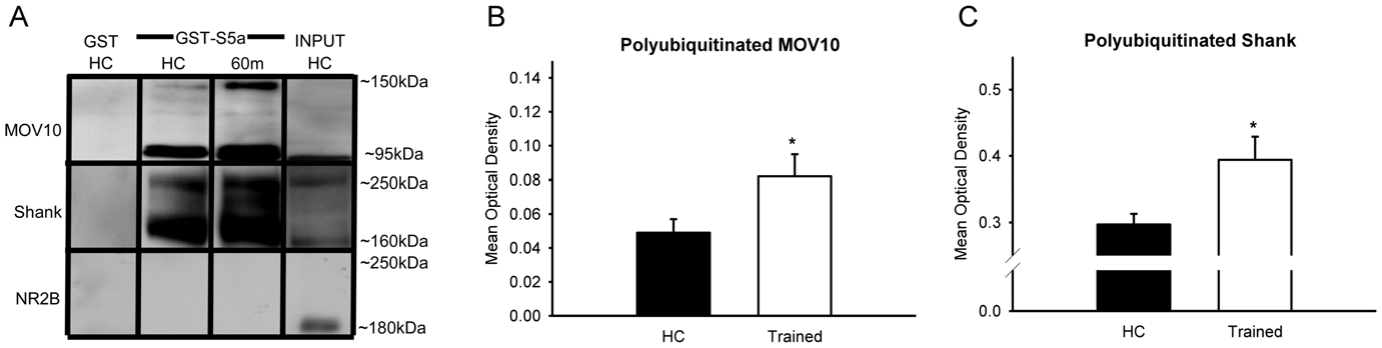
UPS targets proteins involved in translational silencing and synaptic structure in the amygdala. Animals were trained to auditory and context fear conditioning and amygdala tissue was collected 60-min later [n = 10] and compared to naïve homecage [HC] animals [n = 10]. In all cases, crude synaptosomal membrane fractions were obtained, mixed with GST-S5a, and probed with antibodies against MOV10, Shank and NR2B [**A**]. The amount of polyubiquitinated MOV10 [**B**] and Shank [**C]** was increased in trained animals, suggesting potential control over protein synthesis initiation and synaptic restructuring. * denotes p<.05 from HC controls.

We have found that increases in protein degradation following fear conditioning are NMDA dependent and mirror increases in the phosphorylation of kinases involved in translational control. Some in vitro evidence suggests that protein degradation may directly regulate certain forms of protein synthesis and PSD remodeling [16], [21]. We next asked whether UPS activity was involved in the regulation of protein synthesis and PSD remodeling in the amygdala following fear conditioning. Animals were trained with auditory fear conditioning and we collected amygdala crude synaptosomal membrane fractions 60-min later. Since proteolytic-specific polyubiquitination was enhanced only following associative learning [Figure 2], we compared these samples with naïve homecage animals. These samples were purified with GST-S5a and pull-downs were then probed with antibodies against the RISC factor MOV10, PSD scaffolding protein Shank [Figure S2] and PSD receptor protein NR2B [Figure 4A]. ANOVA revealed an increase in the degradation of MOV10 [F_(1, 17)_ = 4.823, p = .042; Figure 4B] and Shank [F_(1, 17)_ = 5.750, p = .028; Figure 4C] following fear conditioning, however, NR2B turnover remained constant. These results generally support previous studies [16], [21], [22] and suggest that following fear conditioning the UPS targets proteins involved in synapse structure and translational silencing indicating that potential functions of the UPS may be a synaptic reorganization process and control over protein synthesis.

### Protein degradation is critical for the formation of long-term fear memories

**Figure 5:**
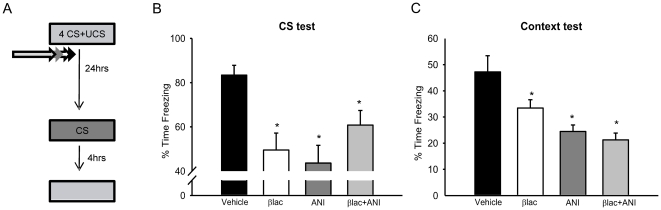
Protein degradation is critical for the formation of long-term fear memories. [**A**] Experimental design for B-C. Animals were trained to auditory and context fear conditioning followed by infusions of βlac [n = 11], ANI [n = 12], βlac+ANI [n = 14] or vehicle [n = 10] into the amygdala. The next day, they were then tested to the auditory cue followed by the context 4-hrs later. [**B, C**] βlac and ANI impaired long-term memory for the auditory and context cues and simultaneous administration of both βlac+ANI did not rescue these impairments. * denotes p<.05 from Vehicle controls.

To more directly test whether UPS activity is critical for long-term memory formation in the amygdala we infused rats with a proteasome inhibitor [βlac] at a dose that rapidly reduces functional proteasome activity [30] [Figure S3] immediately after training [Figure 5A], when memory is normally sensitive to the effects of protein synthesis inhibitors. βlac resulted in significant impairments for both the auditory cue [F_(3,45)_ = 5.32, p = .003] and the context [F_(3,45)_ = 8.735, p<.001] during subsequent drug-free long-term memory tests [Figures 5B and 5C]. Fisher LSD post hoc tests demonstrated that protein degradation blockade resulted in impairments in long-term memory for both cues that was comparable to deficits caused by protein synthesis inhibition [3], [4]. Furthermore, simultaneously blocking both protein degradation and protein synthesis did not rescue these impairments, as has been previously suggested in hippocampal LTP [31]. These results suggest that proteasome-dependent protein degradation is critical for long-term memory formation and plasticity at amygdala synapses.

### Protein degradation is increased in the amygdala following memory retrieval

**Figure 6:**
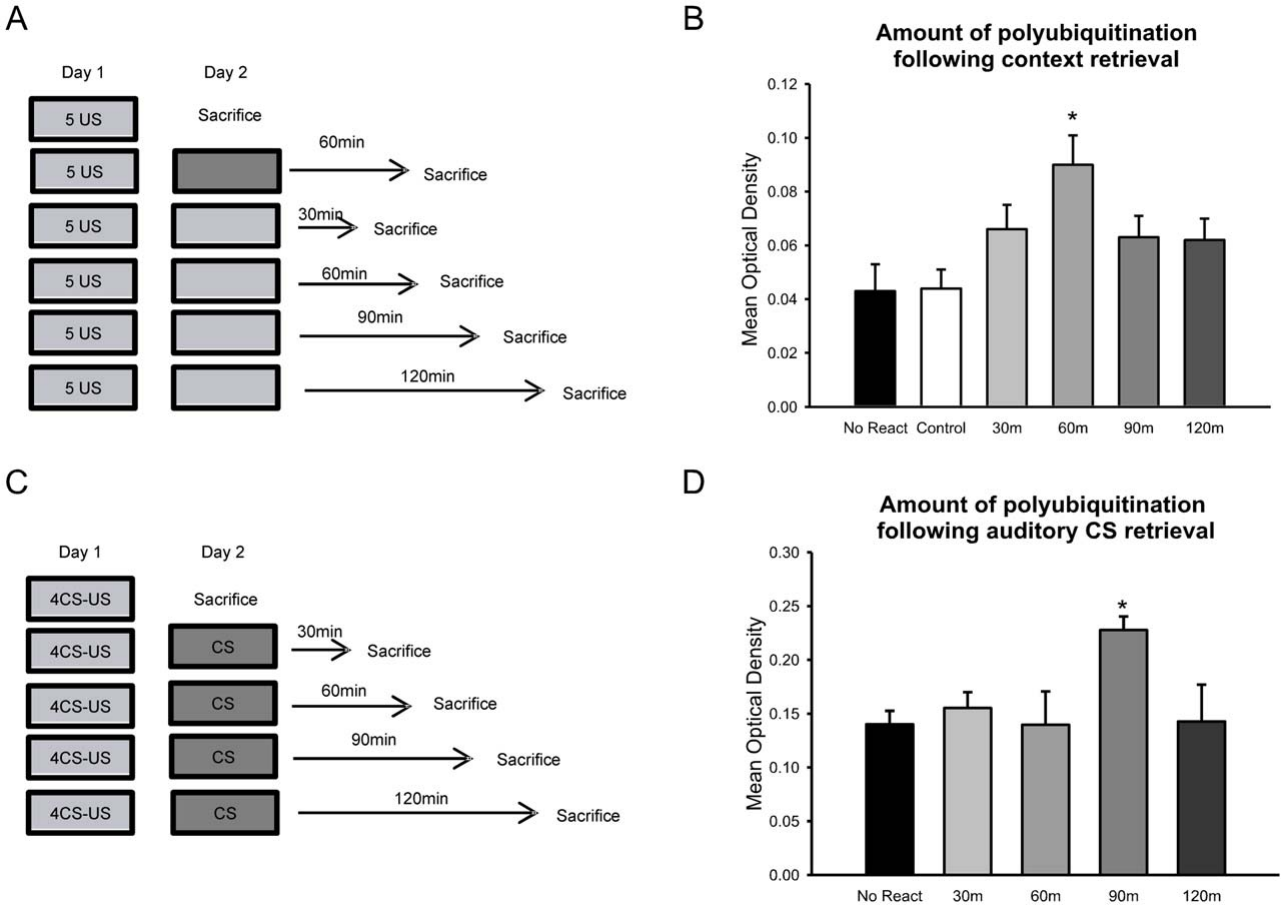
Protein degradation is increased in the amygdala following the retrieval of auditory and context fear memories. [**A**] Experimental design for B. Animals were trained to context fear conditioning on Day 1. The following day, they received a 90-sec reminder to the training context and amygdala tissue was collected in 30-min increments [n = 9 per group]. A separate group of animals was placed into a novel environment and tissue collected 60-min later [n = 7]. Tissue was then purified with GST-S5a. [**B**] There was a rapid increase in the amount of proteins targeted for degradation, which returned to basal levels within 2-hrs. [**C**] Experimental design for D. Animals were trained to auditory fear conditioning on Day 1. The following day, they received a 30-sec reminder to the auditory cue and amygdala tissue was collected in 30-min increments [n = 9-10 per group]. Tissue was then purified with GST-S5a. [**D**] There was a delayed increase in the amount of proteins targeted for degradation, which returned to basal levels within 2-hrs. * denotes p<.05 from controls.

Retrieval of fear memories results in a second phase of protein-synthesis dependence, a process known as memory reconsolidation [8]. Since we found that increases in protein degradation paralleled increases in the activity of proteins involved in translational regulation following acquisition of auditory and context fear conditioning, it may be possible that the retrieval of these same memories will result in increased protein degradation in the amygdala [22]. To investigate this, we trained animals using context fear conditioning, which undergoes a protein synthesis dependent reconsolidation process in the amygdala [32]. The following day, animals received a brief “reminder” exposure to the training context and amygdala tissue was purified with GST-S5a [Figure 6A]. A main effect was found for group during the GST-analysis of protein degradation [F_(5, 46)_ = 3.534, p = .009]. Fisher LSD post hoc tests revealed that protein degradation was rapidly enhanced following retrieval, where it was significantly higher than trained/no retrieval controls at 60-min [Figure 6B]. This increase rapidly returned to basal levels by 90-min after retrieval. Additionally, this increase was due specifically to retrieval in the training context as trained animals treated the same but placed into a novel environment did not show any such enhancements in protein degradation. These results suggest that protein degradation is enhanced in the amygdala following the retrieval of a context fear memory.

Auditory fear memories also undergo a protein synthesis dependent reconsolidation process in the amygdala [3], [8]. To investigate whether protein degradation is increased following retrieval of an auditory fear memory, we trained animals using standard auditory CS/shock UCS pairings. On the following day, the animals were given a brief exposure to the auditory CS in a novel environment after which amygdala tissue was collected and purified with GST-S5a [Figure 6C]. ANOVA revealed a main effect for group following retrieval [F_(4,49)_ = 2.935, p<.030]. Fisher LSD post hoc tests showed that while protein degradation was increased within 60-min of acquisition of auditory fear conditioning and was sustained for at least an hour [Figure 1B], this pattern was more delayed and transient following retrieval of the same fear memory where degradation was significantly higher than controls at 90-min and returned to baseline by 2-hrs [Figure 6D]. This suggests that protein degradation is increased in the amygdala at the same times that protein synthesis is increased, supporting a relationship between the two processes during the reconsolidation of retrieved fear memories.

### The UPS also targets MOV10 and Shank following memory retrieval

**Figure 7:**
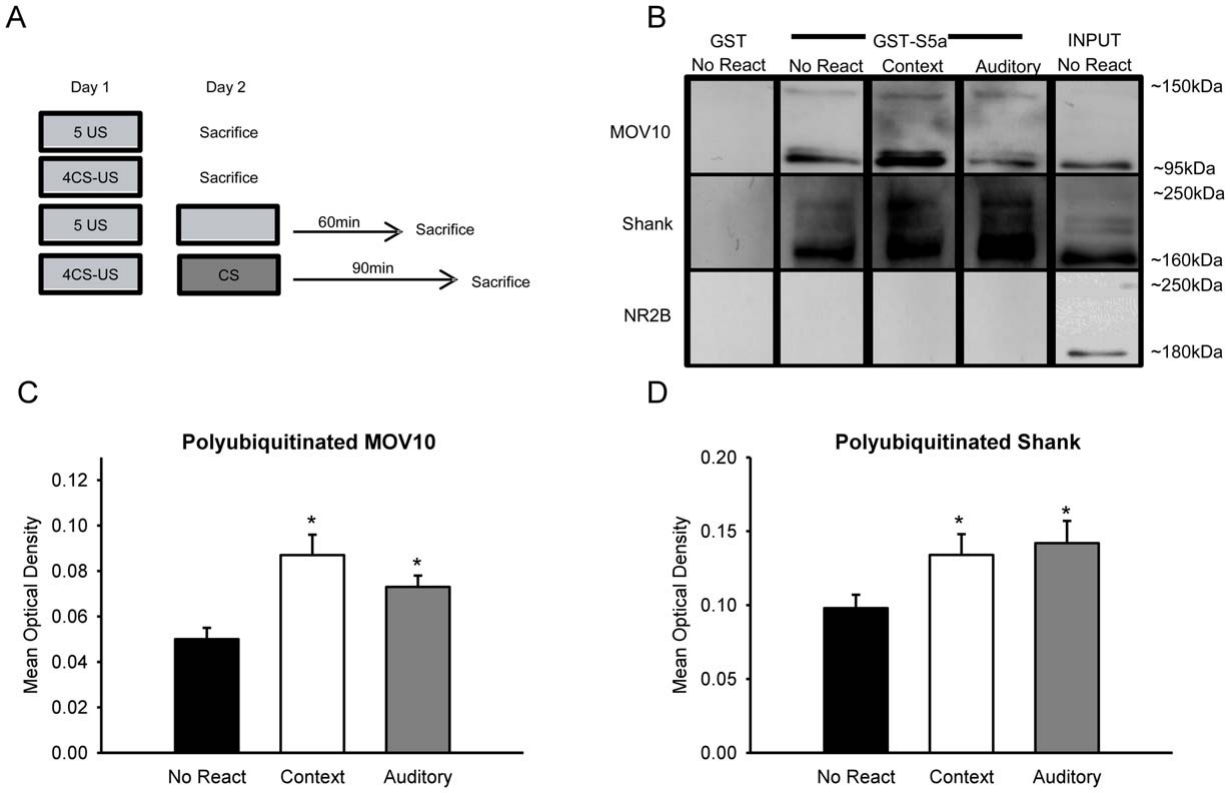
UPS targets proteins involved in translational silencing and synaptic structure in the amygdala following memory retrieval. [**A**] Experimental design for B-D. Animals were trained to auditory or context fear conditioning on Day 1. The next day, animals were exposed to a brief retrieval and amygdala tissue collected 60-min [context, n = 10] or 90-min [auditory, n = 10] later. Two separate groups received auditory or context fear conditioning on Day 1 and were sacrificed on Day 2 without receiving retrieval [n = 6 per group]. Amygdala tissue was fractionated to obtain a crude synaptosomal membrane fraction, purified with GST-S5a, and probed with antibodies against MOV10, Shank and NR2B [**B**]. The amount of polyubiquitinated synaptic MOV10 [**C**] and Shank [**D**], was increased following fear memory retrieval. * denotes p<.05 from No React controls.

To identify what the potential functional role of protein degradation is following memory retrieval we trained animals with auditory or context fear conditioning and collected amygdala tissue at those times at which the peak increases in retrieval-induced protein degradation were noted [Figures 6 and Figure S4] and we fractionated tissue to obtain a crude synaptosomal membrane sample [Figure 7A]. These fractions were then purified with GST-S5a. We found that the UPS targeted MOV10 and Shank but not NR2B following fear conditioning [Figure 3]. To test if the UPS was targeting these same proteins following memory retrieval, pull-downs were then probed with an antibody against MOV10, Shank and NR2B [Figure 7B]. Results indicated a main effect for group on both MOV10 [F_(2, 29)_ = 8.427, p = .001; Figure 7C] and Shank degradation [F_(2, 29)_ = 3.647, p = .039; Figure 7D]. Fisher LSD post hoc tests showed that the degradation of synaptic MOV10 and Shank were significantly increased following both auditory or context fear memory retrieval relative to controls. Again, the turnover rate of NR2B remained constant. These results support previous studies [16], [21], [22] and suggest that the increases in protein degradation in the amygdala following memory retrieval, as well as during the initial memory formation, are at least partially due to the targeting of MOV10 and Shank at amygdala synapses.

### Protein degradation controls the “destabilization” of memory following retrieval

**Figure 8:**
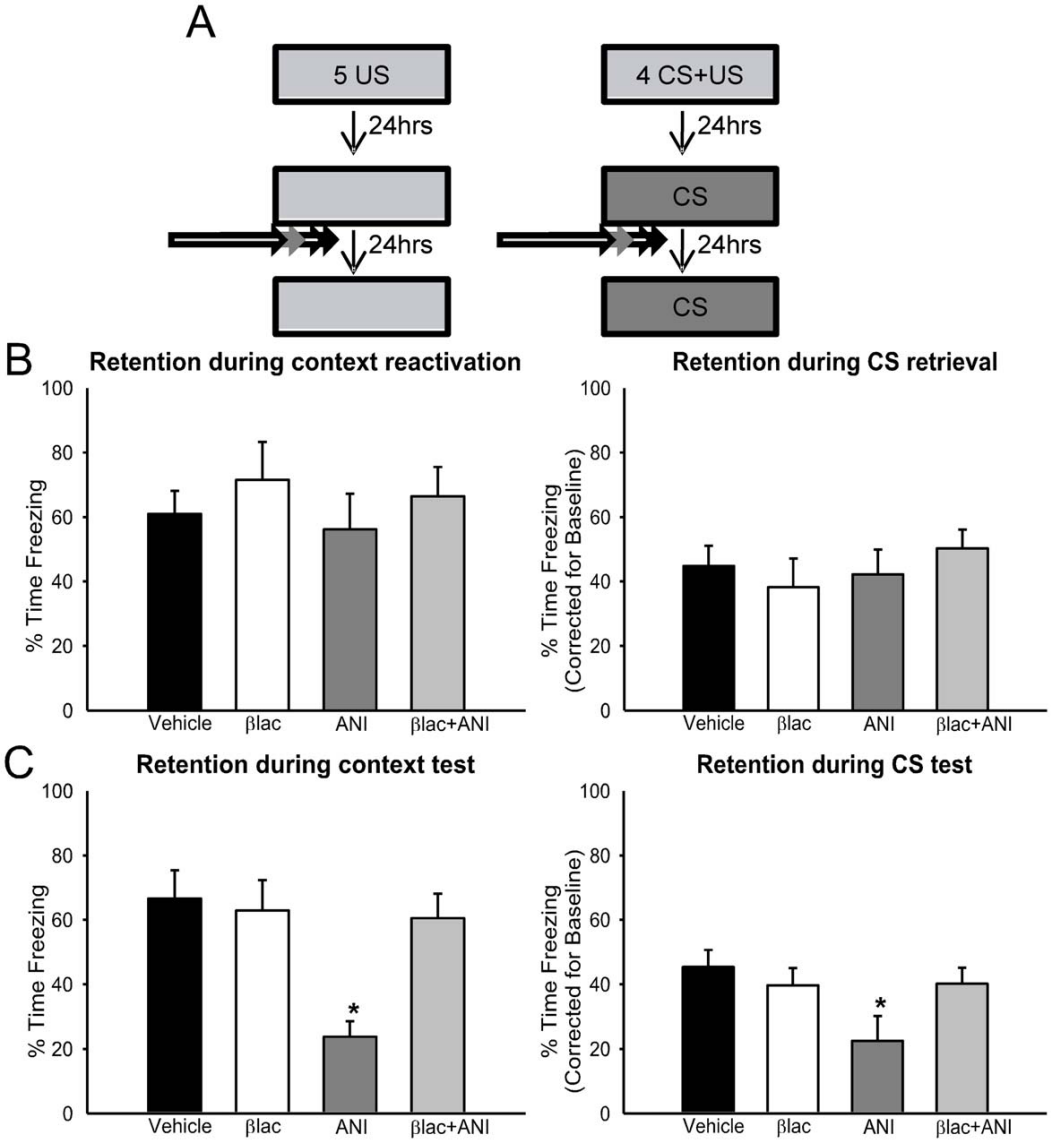
Protein degradation controls the destabilization of retrieved fear memories in the amygdala. [**A**] Experimental design for B-C. Animals were trained to auditory or context fear conditioning on Day 1. The next day, they received a brief retrieval followed by infusions of βlac [auditory n = 13, context n = 7], ANI [auditory n = 12, context n = 7], βlac+ANI [auditory n = 12, context n = 7] or vehicle [auditory n = 13, context n = 7] into the amygdala. The next day, they were then tested to for long-term memory to their acquired cue. [**B**] There were no differences between groups during either context or auditory memory retrieval. [**C**] While βlac had no effect on either memory by itself, it rescued the memory impairments normally caused by ANI when co-infused. * denotes p<.05 from Vehicle controls.

Activation of NMDA receptors is critical for the “destabilization” of retrieved fear memories in the amygdala [33], [34]. When NMDA receptor activity is blocked, the necessity for protein synthesis is removed, and protein synthesis inhibitors are ineffective at disrupting long-term memory. Since we found that the UPS was targeting proteins involved in translational control, this suggests that blocking protein degradation following memory retrieval may prevent the need for new protein synthesis. In order to test this, we trained animals with auditory or context fear conditioning and, on the following day, gave them a brief reminder of the CS. Immediately after the retrieval, animals received an intra-amygdala infusion of the proteasome inhibitor [βlac], the protein synthesis inhibitor anisomycin [ANI], or a combined cocktail [βlac+ANI]. Behavioral performance was assessed on the following day [Figure 8A]. While there were no differences between groups during either context [F_(3, 24)_ = 0.446, p = .722] or auditory [F_(3, 46)_ = 1.110, p = .355] memory retrieval [Figure 8B], there were main effects for both the long-term context [F_(3, 24)_ = 6.540, p = .002] and auditory [F_(3, 46)_ = 2.888, p = .046] CS tests [Figure 8C]. Fisher LSD post hoc tests showed that while blocking protein synthesis alone led to significant impairments in long-term memory for both the contextual and auditory cues, blocking protein degradation by itself did not result in such impairments. Furthermore, simultaneous blockade of protein degradation and synthesis actually prevented impairments normally caused by protein synthesis blockade. This result strongly suggests that changes in protein synthesis are “downstream” of changes in protein degradation during the reconsolidation of auditory and context fear memories in the amygdala.

### Retrieval-induced protein degradation is dependent upon NMDA receptor activity

**Figure 9:**
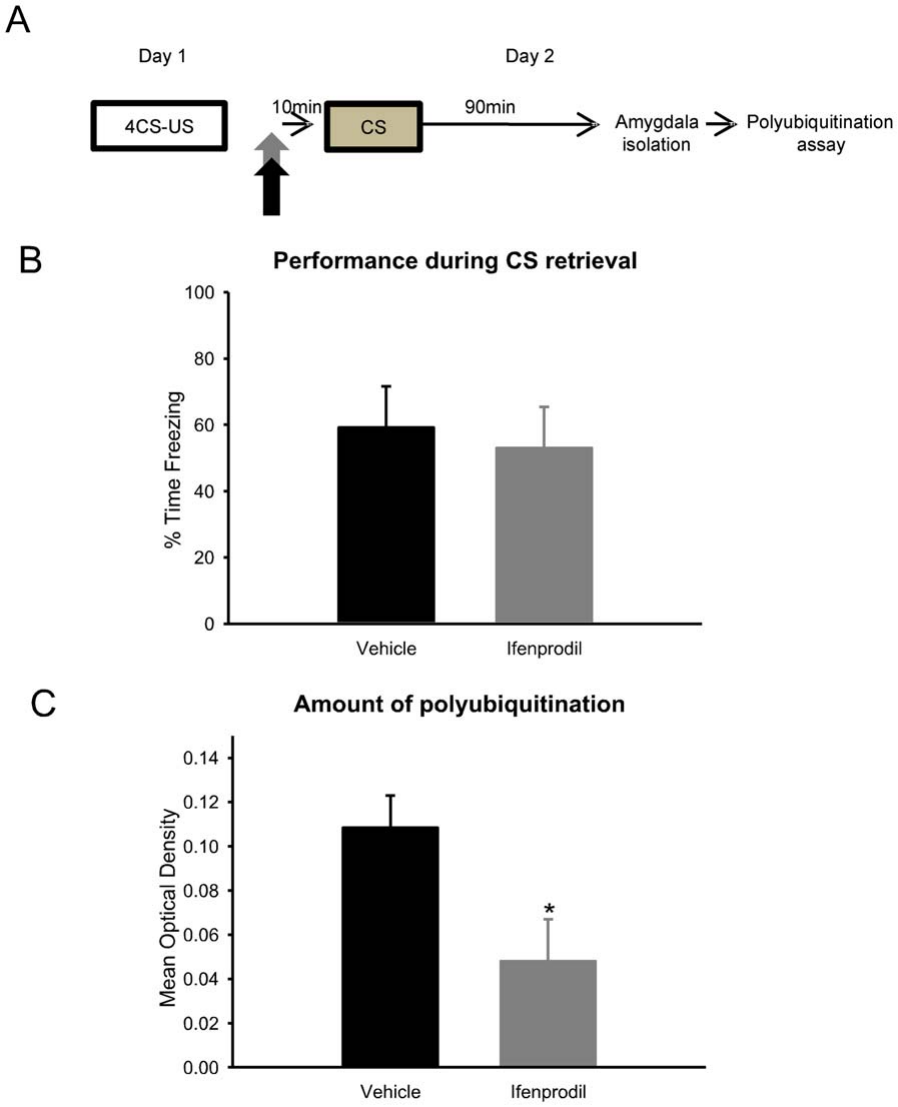
Retrieval-induced increase in protein degradation is dependent on NMDA-receptor activity. (**A**) Infusions of NMDA antagonist Ifenprodil (n = 8) or vehicle (n = 7) were delivered into the amygdala prior to fear memory retrieval, and amygdala tissue collected 90-min later and mixed with GST-S5a. (**B**) Pre-retrieval inactivation of NMDA receptors did not affect retention during CS retrieval, but (**C**) completely impaired the increase in protein degradation. * denotes p<.05 from Vehicle controls.

Blocking NMDA receptors prior to memory retrieval prevents protein synthesis inhibitors from disrupting long-term memory storage [33]. This has led to the idea that activation of NMDA receptors controls the requirement for protein synthesis following memory retrieval [34]. We found that UPS activity was upstream of protein synthesis. Given that NMDA receptor activity appears necessary for increases in protein degradation following acquisition of fear conditioning, we reasoned that there might be a similar requirement following retrieval. To examine this we blocked NMDA receptor activity prior to auditory fear memory retrieval and collected amygdala tissue for GST-analysis [Figure 9A]. Consistent with previous experiments, Ifenprodil significantly reduced the amount of polyubiquitination in the amygdala following retrieval [F_(1, 13)_ = 6.115, p = .028; Figure 9B]. Collectively, these results suggest a pathway whereby NMDA receptor activity signals increases in UPS activity, which controls changes in protein synthesis during the time period following retrieval. This provides the first demonstration of a link between memory destabilization mechanisms at the time of retrieval, which controls changes in protein synthesis necessary for memory updating during the reconsolidation process [7].

## Discussion

Here we present the first comprehensive examination of protein degradation in the formation and stability of long-term memory in the amygdala. We found that protein degradation was rapidly enhanced during the consolidation of fear memories. This increase in degradation was NMDA receptor dependent, paralleled increased translational regulation, and targeted at least two proteins involved in translational silencing and synaptic structure. Furthermore, blocking protein degradation in the amygdala following fear conditioning resulted in significant impairments in long-term memory, suggesting that this process is critical for memory consolidation. Protein degradation was also enhanced in the amygdala following the retrieval of two different fear memories, both of which undergo a protein synthesis dependent reconsolidation process. This increase in degradation following retrieval was more transient than that which followed acquisition, was NMDA-dependent, and also targeted proteins involved in translational control and synaptic structure. Finally, blocking protein degradation after retrieval prevented impairments in long-term memory normally induced by protein synthesis blockade. Collectively, these results suggest that activity-dependent regulation of the UPS is critical in the time period following memory acquisition or retrieval and is necessary for long-term memory storage in the amygdala.

A number of studies have previously suggested that protein synthesis is critical for long-term memory formation in the amygdala [3], [4]. Whether UPS activity is of universal importance in the formation of long-term memory remains to be determined due to a number of conflicting studies using hippocampal-dependent memory tasks [22], [23], [35]. Until now, no study has examined UPS activity in memory formation in the amygdala, the site thought to be critical for the synaptic changes underlying fear conditioning [10]. We found that protein degradation through the UPS was just as important as protein synthesis for memory formation in the amygdala and was likely initiated by a similar mechanism. Furthermore, this increase in protein degradation was related to enhanced polyubiquitination of the synaptic scaffolding protein Shank and RISC factor MOV10, suggesting that protein degradation might be involved in several different aspects of learning-induced synaptic plasticity. What the functional role is of enhanced protein degradation in the amygdala following fear conditioning will be of interest in future studies.

In the present study, we have observed increases in both protein polyubiquitination and phosphorylation following fear conditioning but not following control treatments in which the auditory cue or footshock were presented individually. While this suggests that increased protein degradation and translational regulation may be critical for memory consolidation following the acquisition of a CS-UCS association, it does not rule out the possibility that animals in the control treatments did not learn some information about the auditory cue. It is possible that presentations of the auditory cue by itself could produce learning that is independent of protein degradation and protein synthesis. Consistent with this, recent evidence suggests that unpaired presentations of an auditory cue and footshock do affect synapse size in the lateral amygdala, though not in the same ways that paired presentations do [24]. Thus, in the present study, it is possible that animals receiving auditory cue presentations without footshock did undergo synaptic changes without the protein degradation or protein synthesis alterations that characterize associative learning.

Protein synthesis is critical for the reconsolidation of fear memories following retrieval [8]. Currently, the only mechanism known to control memory “destabilization” or initiate this requirement for protein synthesis in the amygdala is NMDA receptor activity [33], [34]. Our study indicates that protein degradation also controls the need for new protein synthesis in the amygdala following memory retrieval. Of particular interest was the finding that destabilization of context fear memory following retrieval was controlled by UPS activity in the amygdala. Protein degradation has been shown to underlie context memory destabilization in the hippocampus following retrieval [22]. Supporting this, the UPS also targets Shank in both structures following retrieval. Collectively, these results suggest that a retrieved context fear memory is simultaneously destabilized, and “reconsolidated”, in the amygdala and dorsal hippocampus. Whether destabilization in one structure is dependent upon increases in protein degradation in the other structure currently remains unknown. Future research should address this question.

Memory consolidation and reconsolidation share a number of common mechanisms, though some differences have been reported [3], [11], [22], [36]. In the present study, we found that protein degradation was critical for both processes, suggesting that they share UPS activity as a common mechanism. Additionally, in both processes the proteolytic-targeting of MOV10 and Shank was observed. Recent evidence suggests that protein degradation is critically involved in the reconsolidation, but not the consolidation, of context fear memory in the hippocampus [22]. This apparent discrepancy suggests that the amygdala and hippocampus may rely on different mechanisms for initial consolidation, but share similar mechanisms for reconsolidation-related processes. Future research will need to address this in more detail.

Though protein degradation was enhanced following the acquisition and retrieval of fear memories, we found a number of differences in the temporal dynamics of this process. Following acquisition, protein degradation was rapidly increased and sustained for several hours before returning to basal levels by 6-hrs, fitting within the generally understood “consolidation window” [8]. However, following the retrieval of auditory or context fear memory, the increase in protein degradation was more transient and returned to control levels by 2-hrs after stimulus exposure. This supports previous work in suggesting that the reconsolidation process may be shorter than the consolidation process [22]. However, despite this difference in process length, the UPS appeared to target the same proteins. This suggests that differences in mechanisms for memory consolidation and reconsolidation may be due to the speed at which the process occurs. Additionally, the peak in protein degradation in the amygdala occurred at different times following the retrieval of auditory or context fear memories. This discrepancy in the temporal dynamics of protein degradation following retrieval may be due to the influence of other brain regions. For instance, context fear memory depends on hippocampal projections while auditory fear memory depends on projections from the auditory thalamus and auditory cortex [37]–[39]. Furthermore, these projections terminate in different nuclei of the amygdala [40]. In the present study, we did not examine differences in protein degradation between amygdala nuclei. Supporting this, increases in hippocampal protein degradation occur within 60-min of retrieval [22], suggesting that the hippocampus may be influencing the peak time point of degradation in the amygdala following context retrieval.

Learning-induced synaptic plasticity is critical for memory formation and stability in the amygdala. Protein synthesis has long been thought of as a critical component in this process [1]. Here we demonstrate that protein degradation through the UPS is also critically involved. Following both memory acquisition and retrieval, there are NMDA-dependent increases in proteolytic polyubiquitination which are critical for the long-term storage of the memories. Importantly, the UPS appears to target proteins involved in several different processes, including translational control and synaptic structure, suggesting that it may play a substantial role in the consolidation and reconsolidation processes in the amygdala. These results set the framework for future studies to examine the complex role of the UPS in long-term memory formation and stability in amygdala-dependent memory tasks.

## Methods

### Animals

Male Long Evans rats obtained from Harlan [Madison, WI] weighing ∼300–350 grams served as subjects. All animals were housed individually in shoebox cages with free access to water and rat chow. The colony room was maintained under a 14∶10-hr light/dark cycle. Experiments took placed during the light portion of the cycle. All procedures were approved by the University of Wisconsin-Milwaukee Institutional Animal Care and Use Committee (Protocol ID 09-10 #23) and conducted within the ethical guidelines of the National Institutes of Health.

### Surgery

All animals were handled for several days prior to surgery. Rats that underwent surgery were implanted with bilateral cannulas aimed at the amygdala [anteroposterior [AP], −2.8; Lateral [L], +/− 5.0; Ventral [V], −7.2]. Coordinates were chosen based on a rat brain atlas [38]. Before surgery, each rat was anesthetized with an intraperitoneal [IP] injection of sodium pentobarbital [1.5 mg/rat] followed by a second IP injection of ketamine hydrochloride [100 mg/kg]. Animals were then prepared with bilateral stainless steel 26-guage cannulas [Plastics One, Roanoke, VA] which were anchored to the skull using stainless steel screws and acrylic cement. Obdurators [33 gauge] were inserted into the guide cannulae to prevent blockage.

After completion of testing, animals were killed with an intraperitoneal injection of sodium pentobarbital [100 mg/kg]. Animals were transcardially perfused with saline followed by 10% buffered formalin solution. Heads, with cannulas intact, were placed in 10% formalin solution for at least 24 h. The brains were then extracted from the skull and placed in a 20% sucrose formalin solution until they were ready to section. Frozen sections [40 µm] were collected throughout the amygdala, mounted on slides, and stained with cresyl violet. Injection sites were then determined with the aid of a rat brain atlas [41].

### Apparatus

Auditory fear conditioning was conducted in a set of four Plexiglas and stainless-steel observation chambers [Context A] housed in sound-attenuating chambers. The floor was comprised of 18 stainless steel bars 5 mm in diameter spaced 12 mm apart and connected to a shock generator. Ventilation fans produced 62–64 Db of background noise. Each chamber was equipped with a speaker centered in the middle of one end of the chamber. Before testing of each animal, Context A was cleaned with a 5% ammonium hydroxide solution.

Fear to the auditory conditional stimulus [CS] was tested in chambers [Context B] that had floors made of Plexiglas. Fans provided a background noise of ∼58 dB. Each chamber was enclosed in a sound attenuating box and illuminated with a white light. Before testing of each rat, the chambers in Context B were wiped down with a 5% acetic acid solution.

### Drug preparation and infusion procedure

In all cases, rats received bilateral infusions into the amygdala. The total volume of the infusion [0.5 µl/side] was given over 60 s, and the injection cannula remained in place an additional 90 s to ensure diffusion away from the injector tip. The injection cannulae were cut to extend approximately 0.5 mm beyond the guide cannula. Rats were returned to their home cages after infusions. Anisomycin [ANI; 125 µg/µl] and clasto-lactacystin β-lactone [βlac; 32 ng/µl] [both from Sigma, St. Louis, MO] were dissolved in 2% DMSO in HCL, diluted in artificial CSF [aCSF]. A small amount of NaOH was added to bring the pH to ∼7.4. Ifenprodil [Sigma Chemical] was dissolved in DH_2_O. The dosages used were 1 µg/µl for pre-training infusions and 2 µg/µl for pre-retrieval infusions. Theses dosages were determined based on prior research examining fear memory acquisition [28] and retrieval [33] in the amygdala. βlac was prepared as described.

### Behavioral Procedures

One week after surgery, animals received 3 days of acclimation to the transport and handling and injection procedure. Each rat was gently restrained in a towel for several minutes. During this time, the infusion pump to be used during the experiment was activated to habituate the animals to the sound it produces. For experiments using rats without cannulae, animals received 3 days of acclimation to only the transport procedure. There were two training procedures depending upon the experiment. For context fear conditioning, training involved a 2 min baseline followed by five shock [1 mA/1s] presentations separated by a 60 s intertrial interval. After a 2 min postshock period, animals were removed from the training context [Context A]. For auditory fear conditioning, training involved a 6-min baseline followed by four white noise [72dB, 10 s]-shock [1 mA/1s] pairings separated by a 90 s intertrial interval. After a 4 min postshock period, animals were removed from the training context. For the immediate shock condition, animals were placed into Context A and immediately presented with shock and removed. For CS only conditioning, animals were trained to auditory fear conditioning as described above except the shocks were omitted.

Memory retrieval for the auditory CS involved placing the animals in a shifted environment [Context B] and after a 6 min baseline, the animals were provided with a 32 s non-reinforced presentation of the white noise that was paired with shock during training. After a 28 s post-CS period, the animals were removed from the shifted context. Memory retrieval for the context involved placing the animals back into the original training environment [Context A] for 90-sec in the absence of shock. For the drug infusion experiments, animals were given infusions immediately following the end of their training or retrieval session. For posttraining and postretrieval infusions, the animals were removed from the chamber and immediately brought into an adjacent room where they received infusions of βlac, ANI, βlac+ANI or vehicle. Twenty-four hours later, the animals were tested to the auditory and/or context cues. For the auditory test, rats were placed in Context B and after a 6-min baseline, received a 5-min CS presentation in the absence of shock. For the context test, rats were returned to the original conditioning chambers for 8-minutes to assess conditioned fear to the context. Percent time spent freezing during the tests was the measure of learning.

For experiments examining the effects of NMDA-receptor inhibition of protein degradation, animals were trained to auditory fear conditioning as described. For pre-training infusions, animals were infused with Ifenprodil bilaterally into the amygdala 5–10min prior to acquisition. Animals were then trained and tissue collected 60-min later for GST-analysis/immunoblotting. For pre-retrieval infusions, animals were infused with Ifenprodil bilaterally into the amygdala 5–10 min prior to retrieval. Animals were given a retrieval session as described and tissue collected 90-min later for GST-analysis. For quantification of proteasome-inhibition by βlac, naïve animals with bilateral cannulae aimed at the BLA were infused with βlac or vehicle and tissue collected for GST-analysis/immunoblotting 10-, 30-, or 60-min later.

### Conditional fear responses

The activity of each rat was recorded on digital video, and the amount of movement was determined by frame-by-frame changes in pixels using the FreezeScan 1.0 software [Clever Sys, Inc., Reston, VA]. The automatic scoring parameters are chosen such that the scored activity matches hand-scoring methods previously used in this lab to measure freezing. Analyses used percent time spent freezing in response to the CS and context.

### Criterion for exclusion

Rats were excluded from behavioral experiments only if: 1] histological confirmation of cannula placement revealed misplaced cannula on one or both sides of the amygdala or 2] the animals average freezing level was 2 of more standard deviations from the group mean.

### Whole cell tissue preparation

Amygdala tissue was dissected out by blocking the brain in a rat brain matrix [Harvard Apparatus, Holliston, MA] and making a single coronal cut at the anterior tip of the amygdala and one at the posterior end of the amygdala. Both sides of the whole amygdala were dissected out from the blocked tissue by making a cut along the external capsule and a diagonal cut along the optic tract. The tissue sample was homogenized in buffer [all in 100 ml DDH20; 0.605 g Tris-HCl, 0.25 g sodium deoxycholate, 0.876 g NaCl, 0.038 g EDTA, 0.0042 g NaF, 1 µg/ml PMSF, 1 µg/ml leupeptin, 1 µg/ml aprotinin, 10 ml 10% SDS, 1 Mm sodium orthovanadate] and immediately placed on dry ice. Samples were stored at −80°C until needed. Samples were thawed and then centrifuged at 4000 rpm for 20 min at 4°C; the supernatant was removed and measured using a Bradford protein assay kit [BioRad, Hercules, CA].

### Synaptosomal membrane preparation

The amygdala was dissected out as described above. Crude synaptosomal membrane fractions were obtained as described previously with a small scale modification [42]. Briefly, samples were homogenized in TEVP with 320 mM sucrose and centrifuged at 1000 x g for 10-min, 4°C. The supernatant was collected and centrifuged at 10,000 x g for 10-min, 4°C. The resulting pellet was denatured in Lysis buffer [all in 100 ml DDH20; 0.605 g Tris-HCl, 0.25 g sodium deoxycholate, 0.876 g NaCl, 1 µg/ml PMSF, 1 µg/ml leupeptin, 1 µg/ml aprotinin, 10 ml 10% SDS] and centrifuged at 15,000 x g for 5-min, 4°C. The supernatant was collected and measured using a Bradford protein assay kit [BioRad, Hercules, CA].

### GST-Pull Down

For GST-Pull Downs, 25- [synaptosomal] or 50-µg [whole cell] of each sample were diluted in a TBS Wash Buffer [25 Mm Tris, 75 Mm NaCl, 5% Glycerol, 1% Triton, 1 µg/ml PMSF, 1 µg/ml aprotinin, pH 7.5]. These diluted samples were then mixed with GST-S5a agarose [Enzo Life Sciences, Plymouth Meeting, PA, USA] or an equivalent amount of GST-agarose. Samples were then incubated for 2hrs at 4°C. Following incubation, samples were centrifuged at 500 x *g* and the supernatant collected. All samples were then extensively washed in TBS Wash Buffer and boiled in SDS-loading buffer at 95°C for 4-min. Following boiling, samples were briefly centrifuged at 500 x *g* and the supernatant collected.

### Western Blotting

Samples were loaded on 5–9% SDS-PAGE. Proteins were transferred from the gel to a membrane using a semidry transfer apparatus [Bio-Rad]. Membranes were incubated in blocking buffer for 1-hr and then incubated overnight at 4°C in primary antibody for ubiquitin, NR2B, [both 1∶1000; Cell Signaling, Danvers, MA, USA], phospho-mTOR, mTOR [both 1∶500, Cell Signaling], phospho-P70S6K, P70S6K, K48 polyubiquitin [all 1∶1000, Chemicon], Shank, [1∶500; NeuroMab, Irvine, CA, USA] or MOV10 [1∶500; Bethyl Laboratories, Montgomery, TX, USA]. After primary antibody exposure, the membranes were incubated in secondary antibody [dilution 1∶2000 – 1∶5000; Santa Cruz Biotechnology, Santa Cruz, CA, USA] for 60-min. Membranes were washed thoroughly, placed in a chemiluminescence solution for 3-min [Santa Cruz Biotechnology], and exposed to autoradiographic film [Hyperfilm MP]. Images were taken and densitometry performed using NIH Image J. For ubiquitin, optical density was taken from all captured proteins along the entire molecular standards ladder.

### Statistical analysis

For behavioral experiments, the average percent time spent freezing was calculated for each group. For the auditory retrieval experiment, due to differences in the success of the context shift at the time of retrieval, the average time spent freezing during the baseline period was subtracted away from the average time spent freezing during the CS period for both the retrieval and testing sessions. This was done to equate groups prior to drug treatment. For quantitative protein assays, mean optical densities were calculated for each group. Data was analyzed using Analysis of Variance [ANOVA]. Fisher Least Significant Differences [LSD] *post hoc* testes were used where appropriate.

## Supporting Information

Figure S1 **Fear conditioning increases the amount of K48 linked polyubiquitinated proteins.** (**A**) Animals were presented with either 4 pairings of the auditory cue with shock or 4 presentations of the auditory cue by itself. Only animals receiving pairings of the stimuli showed fear to the auditory cue (CS-UCS presentations) and the context (Post CS period). (**B**) Samples were ran on 7.5% SDS-PAGE and developed against K48 polyubiquitin. K48 polyubiquitination was increased only 60-min after fear conditioning. (**C, D**) There were no changes in total P70S6 kinase or total mTOR. (**E**) There were no differences in β-actin, which was used as a loading control. * denotes p<.05.
(TIF)

Figure S2 **Antibody recognizes a distinct band for Shank at ∼160 kDa.** 50 µg of amygdala whole cell lysate was loaded on 5% gels and exposed to an antibody against Shank. The antibody recognized a distinct band at 160kDa (Shank 1), as well as some alternative splicing products.
(TIF)

Figure S3 **βlac results in a rapid and persistent accumulation of polyubiquitinated proteins.** Naïve animals were infused with βlac into the amygdala and tissue collected 10- (n = 6), 30- (n = 6) or 60-min (n = 6) later. Separate animals were infused with vehicle (n = 6). (**A**) Samples were purified with GST-S5a. βlac resulted in a rapid and persistent accumulation of polyubiquitinated proteins in the amygdala (F_(3, 21)_ = 5.876, p = .004), suggesting effective inhibition of proteasome activity. (**B**) Much of this protein accumulation was due to inhibited degradation of K48-linked polyubiquitinated proteins (*F*_(3, 21)_ = 4.576, *P* = .013). * denotes p<.05 from Vehicle controls.
(TIF)

Figure S4 **Synaptic protein degradation is increased following fear memory retrieval.** Animals were trained with auditory or context fear conditioning and amygdala tissue collected 60- or 90-min later. Tissue was fractionated to obtain a crude synaptosomal membrane sample and these fractions were then purified with GST-S5a. A main effect for group was found for the amount of polyubiquitination following retrieval (F_(2, 29)_ = 3.459, p = .045). * denotes p<.05 from No React controls.
(TIF)

## References

[1] Davis HP, Squire LR (1984). Protein synthesis and memory: a review.. Psycho Bull.

[2] Bailey DJ, Kim JJ, Sun W, Thompson RF, Helmstetter FJ (1999). Acquisition of fear conditioning in rats requires the synthesis of mRNA in the amygdala.. Behav Neurosci.

[3] Parsons RG, Gafford GM, Baruch DE, Riedner BA, Helmstetter FJ (2006). Long-term stability of fear memory depends on the synthesis of protein but not mRNA in the amygdala.. Eur J Neurosci.

[4] Schafe GE, LeDoux JE (2000). Memory consolidation of auditory pavlovian fear conditioning requires protein synthesis and protein kinase A in the amygdala.. J Neurosci.

[5] Helmstetter FJ, Parsons RG, Gafford GM (2008). Macromolecular synthesis, distributed synaptic plasticity, and fear conditioning.. Neurobiol Learn Mem.

[6] McGaugh JL (2000). Memory—a century of consolidation.. Science.

[7] Lee JLC (2008). Memory reconsolidation mediates the strengthening of memory by additional learning.. Nat Neurosci.

[8] Nader K, Schafe GE, LeDoux JE (2000). Fear memories require protein synthesis in the amygdala for reconsolidation following retrieval.. Nature.

[9] Sara SJ (2000). Retrieval and reconsolidation: towards a neurobiology of remembering.. Learn Mem.

[10] Fanselow MS, LeDoux JE (1999). Why we think plasticity underlying Pavlovian fear conditioning occurs in the basolateral amygdala.. Neuron.

[11] Tronson NC, Taylor JR (2007). Molecular Mechanisms of Reconsolidation.. Nat Rev Neurosci.

[12] Parsons RG, Gafford GM, Helmstetter FJ (2006). Translational control via the mammalian target of rapamycin pathway is critical for the formation and stability of long-term fear memory in amygdala neurons.. J Neurosci.

[13] Hershko A, Ciechanover A (1998). The Ubiquitin System.. Annu Rev Biochem.

[14] Layfield R, Tooth D, Landon M, Dawson S, Mayer J (2001). Purification of poly-ubiquitinated proteins by S5a-affinity chromatography.. Proteomics.

[15] Bedford L, Paine S, Sheppard PW, Mayer RJ, Roelofs J (2010). Assembly, structure and function of the 26S proteasome.. Trends Cell Biol.

[16] Ehlers MD (2003). Activity level controls postsynaptic composition and signaling via the ubiquitin-proteasome system.. Nat Neurosci.

[17] Hegde, AN (2010). The ubiquitin-proteasome pathway and synaptic plasticity.. Learn Mem.

[18] Mabb AM, Ehlers MD (2010). Ubiquitination in postsynaptic function and plasticity.. Annu Rev Cell Dev Biol.

[19] Tai HC, Schuman EM (2008). Ubiquitin, the proteasome and protein degradation in neuronal function and dysfunction.. Nat Rev Neurosci.

[20] Bingol B, Sheng M (2010). Deconstruction for reconstruction: the role of proteolysis in neural plasticity and disease.. Neuron.

[21] Banerjee S, Neveu P, Kosik KS (2009). A coordinated local translational control point at the synapse involving relief from silencing and MOV10 degradation.. Neuron.

[22] Lee S-H, Choi J-H, Lee N, Lee H-R, Kim J-I (2008). Synaptic protein degradation underlies destabilization of retrieved fear memory.. Science.

[23] Lopez-Salon M, Alonso M, Vianna MR, Viola H, Mello e Souza T (2001). The ubiquitin-proteasome cascade is required for mammalian long-term memory formation.. Eur J Neurosci.

[24] Ostroff LE, Cain CK, Bedont J, Monfils MH, LeDoux JE (2010). Fear and safety learning differentially affect synapse size and dendritic translation in the lateral amygdala.. Proc Natl Acad Sci U S A.

[25] Bingol B, Wang CF, Arnott D, Cheng D, Peng J (2010). Autophosphorylated CaMKIIalpha acts as a scaffold to recruit proteasomes to dendritic spines.. Cell.

[26] Newton K, Matsumoto ML, Wertz IE, Kirkpatrick DS, Lill JR (2008). Ubiquitin chain editing revealed by polyubiquitin linkage-specific antibodies.. Cell.

[27] Wang Q, Young P, Walters KJ (2005). Structure of S5a bound to monoubiquitin provides a model for polyubiquitin recognition.. J Mol Biol.

[28] Rodrigues SM, Schafe GE, LeDoux JE (2001). Intra-amygdala blockade of the NR2B subunit of the NMDA receptor disrupts the acquisition but not the expression of fear conditioning.. J Neurosci.

[29] Bingol B, Schuman EM (2006). Activity-dependent dynamics and sequestration of proteasomes in dendritic spines.. Nature.

[30] Rinetti GV, Schweizer FE (2010). Ubiquitination acutely regulates presynaptic neurotransmitter release in mammalian neurons.. J Neurosci.

[31] Fonseca R, Vabulas RM, Hartl FU, Bonhoeffer T, Nagerl UV (2006). A balance of protein synthesis and proteasome-dependent degradation determines the maintenance of LTP.. Neuron.

[32] Mamiya N, Fukushima H, Suzuki A, Matsuyama Z, Homma S (2009). Brain region-specific gene expression activation required for reconsolidation and extinction of contextual fear memory.. J Neurosci.

[33] Mamou CB, Gamache K, Nader K (2006). NMDA receptors are critical for unleasing consolidated auditory fear memories.. Nat Neurosci.

[34] Wang SH, de Oliveira Alvares L, Nader K (2009). Cellular and systems mechanisms of memory strength as a constraint on auditory fear reconsolidation.. Nat Neurosci.

[35] Artinian J, McGauran AM, De Jaeger X, Mouledous L, Frances B (2008). Protein degradation, as with protein synthesis, is required not only during long-term spatial memory consolidation but also reconsolidation.. Eur J Neurosci.

[36] Lee JL, Everitt BJ, Thomas KL (2004). Independent cellular processes for hippocampal memory consolidation and reconsolidation.. Science.

[37] Kim JJ, Fanselow MS (1992). Modality-specific retrograde amnesia of fear.. Science.

[38] Phillips RG, LeDoux JE (1992). Differential contribution of the amygdala and hippocampus to cued and contextual fear conditioning.. Behav Neurosci.

[39] Romanski LM, LeDoux JE (1992). Equipotentiality of thalamo-amygdala and thalamo-cortico-amygdala circuits in auditory fear conditioning.. J Neurosci.

[40] LeDoux JE (2000). Emotion circuits in the brain.. Annu Rev Neurosci.

[41] Paxinos G, Watson C (1998). The rat brain in stereotaxic coordinates: Edition 4..

[42] Dunah AW, Standaert DG (2001). Dopamine D1 receptor-dependent trafficking of Striatal NMDA glutamate receptors to the postsynaptic membrane.. J Neurosci.

