# Supplementary figures and images for: Activity Dependent Protein Degradation Is Critical for the Formation and Stability of Fear Memory in the Amygdala
Source: PLoS One. 2011 Sep 22;6(9):e24349. doi: 10.1371/journal.pone.0024349 (PMC3178530; doi:10.1371/journal.pone.0024349)

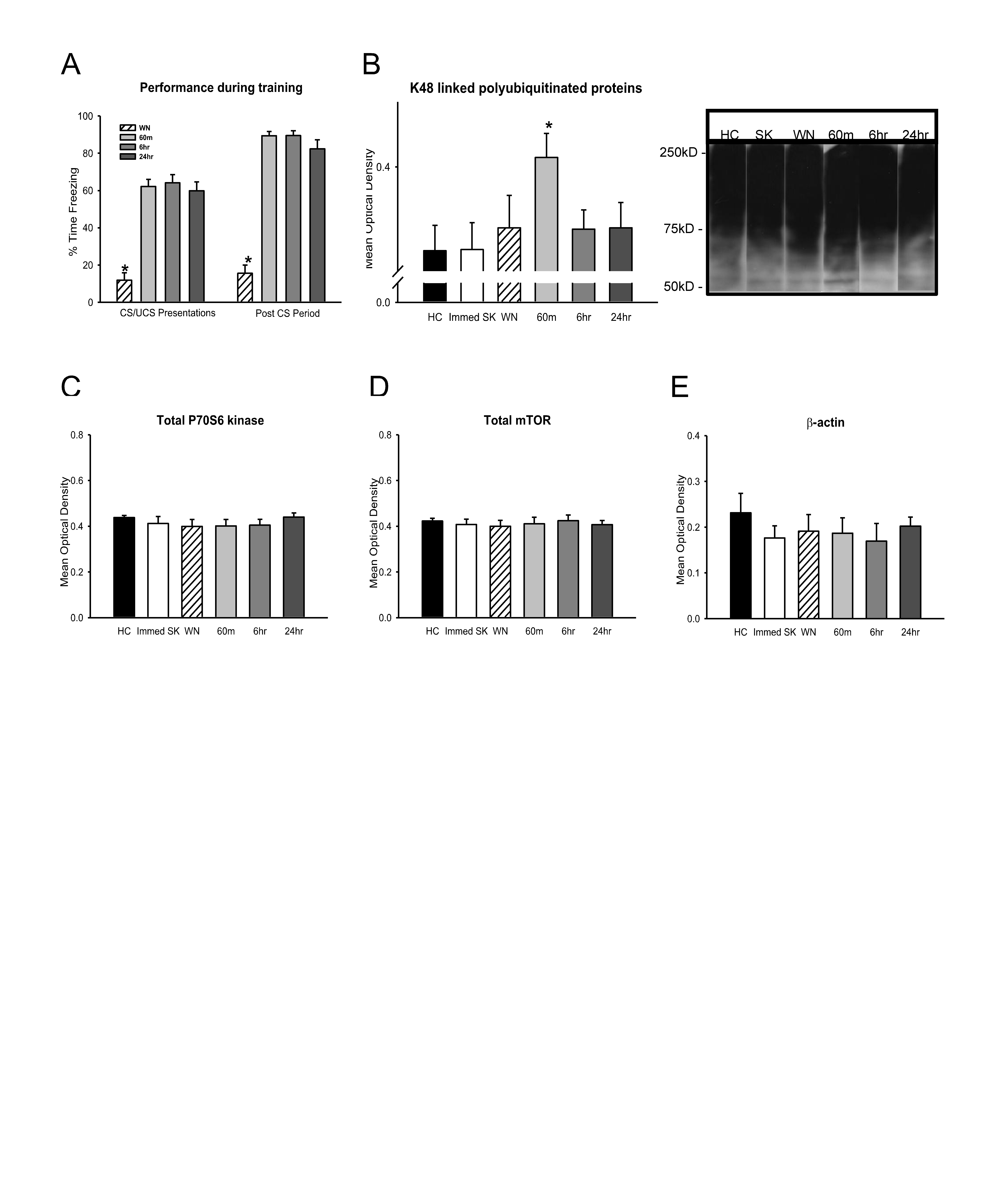

Supplement: Figure S1 — Fear conditioning increases the amount of K48 linked polyubiquitinated proteins. (A) Animals were presented with either 4 pairings of the auditory cue with shock or 4 presentations of the auditory cue by itself. Only animals receiving pairings of the stimuli showed fear to the auditory cue (CS-UCS presentations) and the context (Post CS period). (B) Samples were ran on 7.5% SDS-PAGE and developed against K48 polyubiquitin. K48 polyubiquitination was increased only 60-min after fear conditioning. (C, D) There were no changes in total P70S6 kinase or total mTOR. (E) There were no differences in β-actin, which was used as a loading control. * denotes p<.05. (TIF) [file pone.0024349.s001.tif]

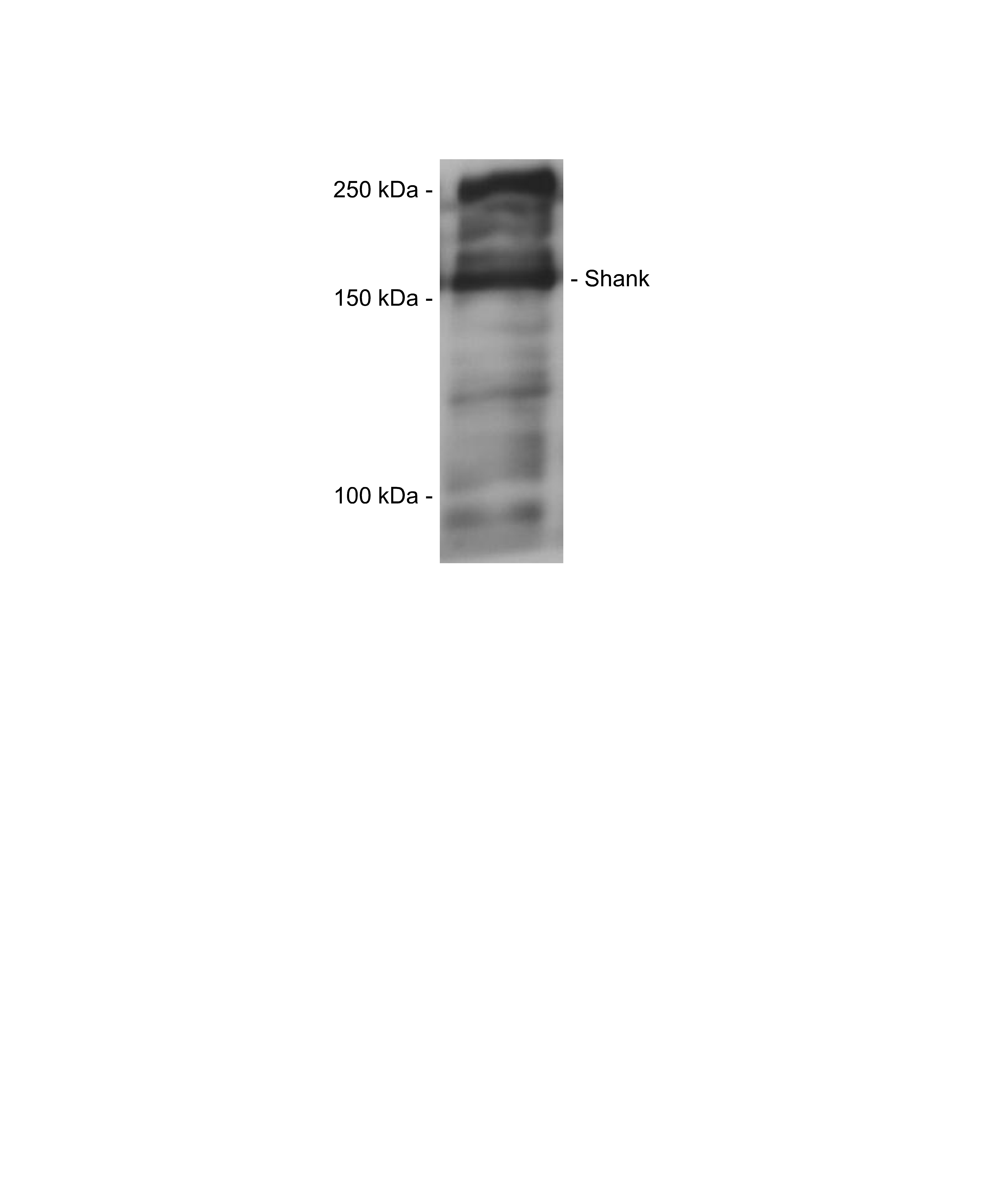

Supplement: Figure S2 — Antibody recognizes a distinct band for Shank at ∼160 kDa. 50 µg of amygdala whole cell lysate was loaded on 5% gels and exposed to an antibody against Shank. The antibody recognized a distinct band at 160kDa (Shank 1), as well as some alternative splicing products. (TIF) [file pone.0024349.s002.tif]

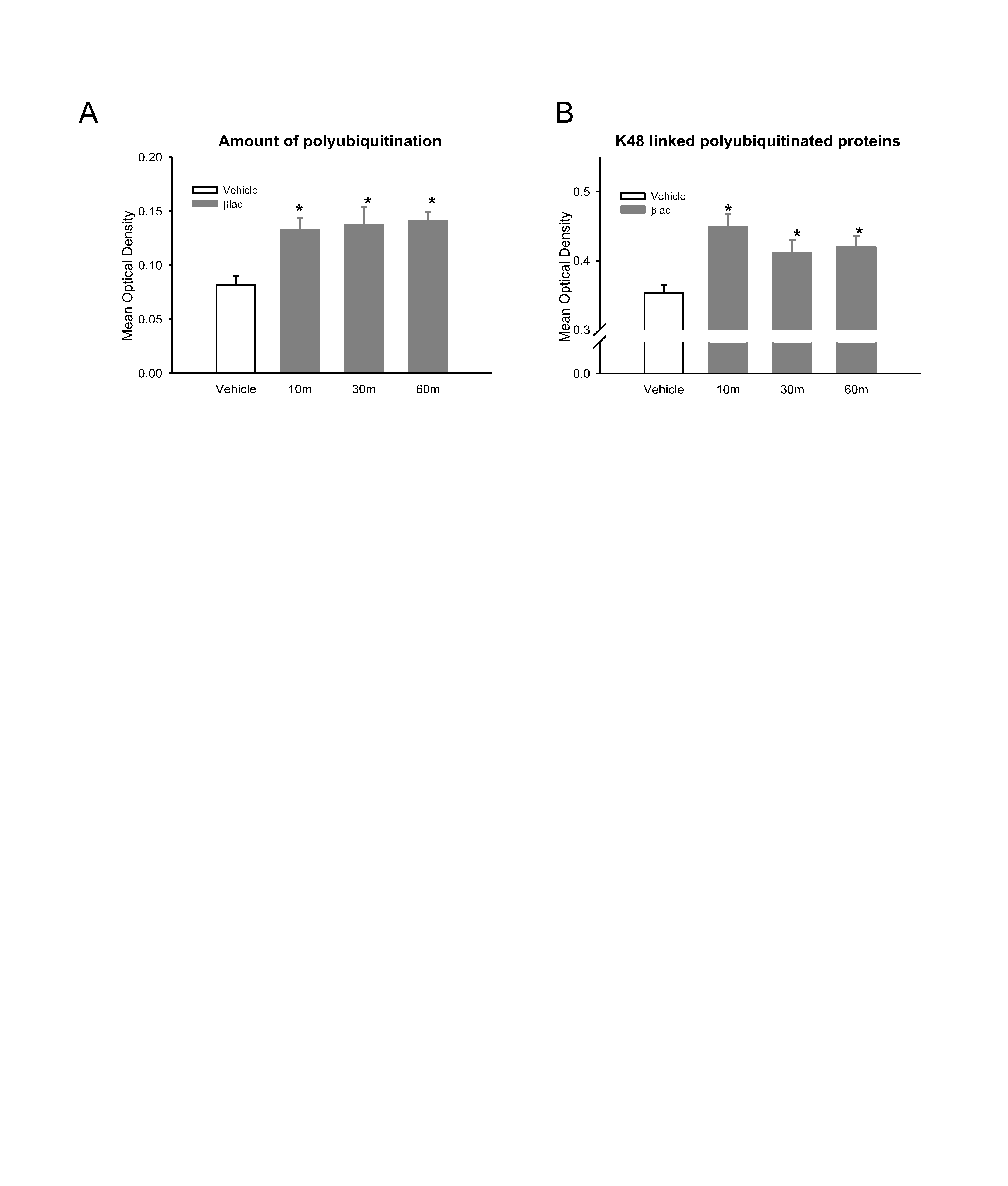

Supplement: Figure S3 — βlac results in a rapid and persistent accumulation of polyubiquitinated proteins. Naïve animals were infused with βlac into the amygdala and tissue collected 10- (n = 6), 30- (n = 6) or 60-min (n = 6) later. Separate animals were infused with vehicle (n = 6). (A) Samples were purified with GST-S5a. βlac resulted in a rapid and persistent accumulation of polyubiquitinated proteins in the amygdala (F(3, 21) = 5.876, p = .004), suggesting effective inhibition of proteasome activity. (B) Much of this protein accumulation was due to inhibited degradation of K48-linked polyubiquitinated proteins (F (3, 21) = 4.576, P = .013). * denotes p<.05 from Vehicle controls. (TIF) [file pone.0024349.s003.tif]

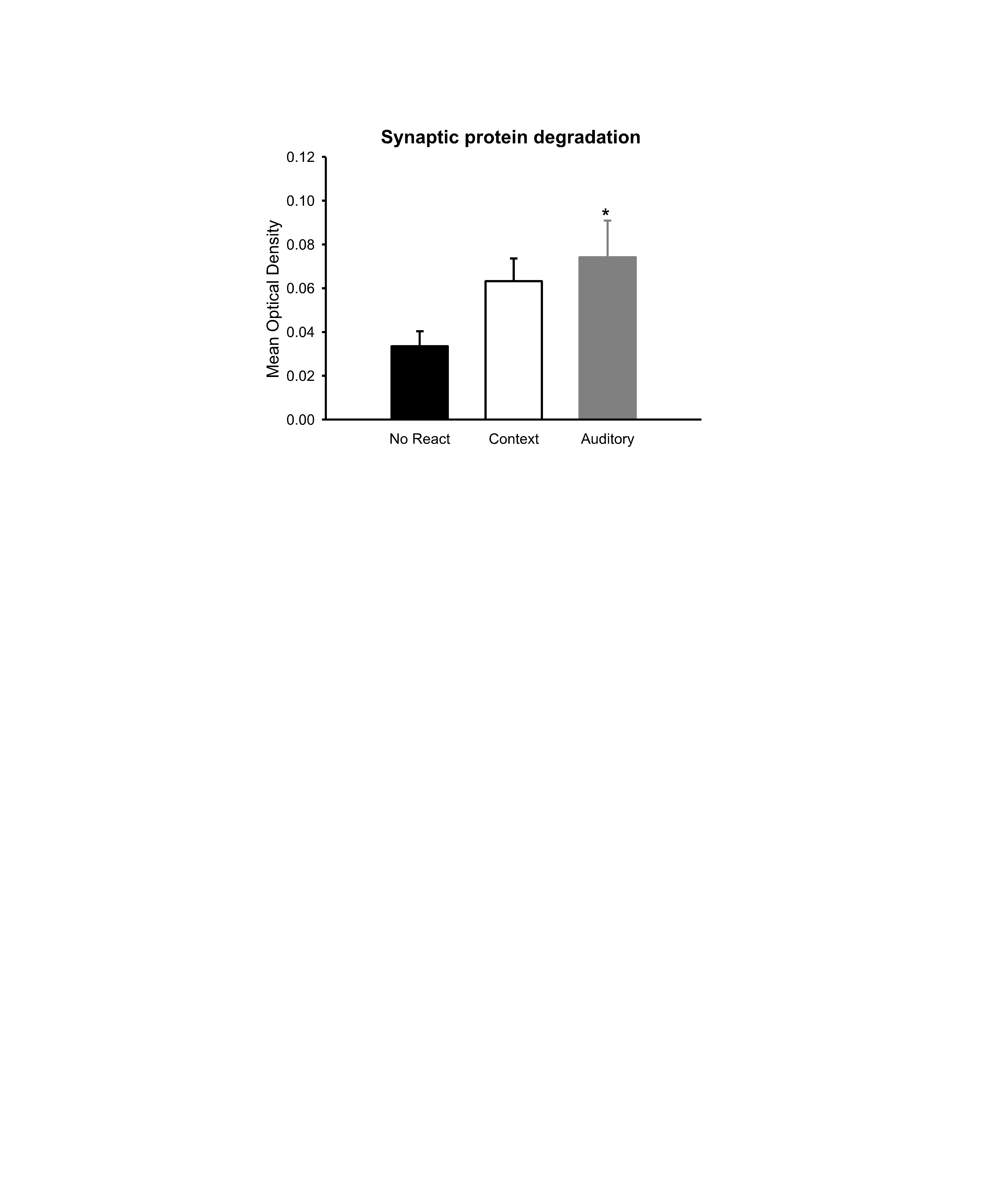

Supplement: Figure S4 — Synaptic protein degradation is increased following fear memory retrieval. Animals were trained with auditory or context fear conditioning and amygdala tissue collected 60- or 90-min later. Tissue was fractionated to obtain a crude synaptosomal membrane sample and these fractions were then purified with GST-S5a. A main effect for group was found for the amount of polyubiquitination following retrieval (F(2, 29) = 3.459, p = .045). * denotes p<.05 from No React controls. (TIF) [file pone.0024349.s004.tif]
